# Postural regulation and stability with acoustic input in normal-hearing subjects

**DOI:** 10.1007/s00106-020-00846-9

**Published:** 2020-05-06

**Authors:** I. Seiwerth, J. Jonen, T. Rahne, A. Lauenroth, T. E. Hullar, S. K. Plontke, R. Schwesig

**Affiliations:** 1 Department of Otorhinolaryngology, Head & Neck Surgery, Martin Luther University Halle-Wittenberg, University Medicine Halle (Saale), Halle (Saale), Germany; 2 Department of Orthopedics and Trauma Surgery, Martin Luther University Halle-Wittenberg, University Medicine Halle (Saale), Halle (Saale), Germany; 3 School of Medicine, Department of Otolaryngology—Head and Neck Surgery, Oregon Health & Science University, Portland, OR USA

**Keywords:** Posturography, Musculoskeletal physiological phenomena, Posture, Hearing, Postural balance

## Abstract

**Background:**

Postural regulation is based on complex interactions among postural subsystems. The auditory system too appears to have an influence on postural control.

**Objective:**

The aim of this study was to measure the influence of auditory input on postural control and to gain a deeper understanding of the interactions between auditory input and postural subsystems including subjective aspects.

**Materials and methods:**

In 30 healthy normal-hearing subjects, postural regulation and stability was measured with the Interactive Balance System (IBS; Inc. neurodata GmbH, Wien, Österreich) in 8 test positions with noise (frontal presentation) and plugged without noise. The IBS is an electrophysiological measurement device that measures postural control at the product level (e.g., stability, weight distribution) and the mechanisms of postural subsystems at the process level based on frequency-oriented fast-Fourier analysis of force–time relation.

**Results:**

At the process level, we found a relevant reduction (η_p_^2^ ≥ 0.10) of postural regulation with noise in the frequency bands F1 (visual and nigrostriatal system η_p_^2^ = 0.122) and F2–4 (peripheral vestibular system η_p_^2^ = 0.125). At the product level, the weight distribution index (WDI) parameter showed a relevant increase with noise (η_p_^2^ = 0.159). No difference between the auditory conditions was found for postural stability (parameter: stability indicator, ST). Substantial interindividual variations in the subjective estimation of the influence of auditory inputs on stability were observed.

**Conclusion:**

In this study, a shift in the activity of postural subsystems was observed with auditory input, while no difference was seen in ST. This leads to new insights into mechanisms of audiovestibular interaction.

## Background

Postural regulation is based on complex interaction mechanisms of postural subsystems. It has been shown several times in healthy subjects [5, 8, 9, 11, 17, 18, 28, 31, 33, 35] as well as in patients with hearing amplification [10, 19, 29, 30, 33, 34] that the auditory system also plays a role in addition to visual, proprioceptive, and vestibular cues. In the majority of studies, a positive influence of hearing on balance and postural stability has been reported. However, in some studies, no influence of auditory input on balance was described [2, 4, 5, 12, 13], while other studies reported a destabilizing effect [4, 14, 32]. The studies often differ considerably with regard to the subject group, the posturography method, as well as the character and method of presentation of noise.

In most of the established measurement methods, validation of postural regulation or stability is performed by using parameters that are based on the execution or the performance of defined procedures or testing situations. For example, the gait deviation in the Unterberger (Fukuda) stepping test, pressure changes in footplate measurement systems, or trunk sway in gait analysis are indicators of postural control. The ability to maintain the correct position and orientation of the body in space is the result (product) of complex regulatory mechanisms of previously described subsystems (processes).

Sole consideration of the product level is often not sufficient for knowing how these subsystems interact and which hierarchical role they play depending on the respective situation, even if some conclusions can be drawn indirectly from the product level.

The Interactive Balance System (IBS) is a force plate system that measures parameters of the product level such as stability or force changes between heel and forefoot and provides insights into the working mechanisms of postural subsystems (process level) based on frequency-oriented fast Fourier analysis of force as a function of time. Accordingly, each postural subsystem corresponds to a respective frequency range, which has been validated in several studies: For example, the frequency band F1 (0.03–0.1 Hz) has been associated with the visual system by comparative studies on visually impaired and people with normal vision [7, 21]. Similarly, the frequency ranges of the nigrostriatal, the cerebellar, the peripheral vestibular, and the somatosensory system were validated in patients with Parkinson’s disease (nigrostriatal; [23]), patients with cerebellar disorders ([23]; cerebellar), patients wearing cochlear implants [21], patients with vestibular neuritis ([24]; peripheral vestibular), and in tests with plantar cold application ([21]; somatosensory).

Furthermore, the IBS has already been tested several times with respect to reliability [22, 24, 27] and has facilitated the evaluation of influencing factors on postural stability such as the cerebellar and nigrostriatal system [23], the visual system [7, 20], and age-related changes [25].

The aim of this prospective experimental study was to verify the hypothesis that acoustic input is an influencing factor on postural control. Furthermore, we expected to gain more in-depth information on the interaction mechanisms of subsystems contributing to postural regulation.

## Material and methods

The study was carried out with normal-hearing, healthy subjects. Inclusion criteria were age of 18–70 years, a body mass index (BMI) of <30, normal hearing based on DIN ISO 7029 (4PTA_0.5–4_ _kHz_), and the lack of subjectively perceived or objective vertigo. Exclusion criteria were the influence of medication affecting the vestibular system, alcohol and drugs, as well as any physical limitation.

### Method

To qualify for inclusion criteria, an examination of the tympanic membrane as well as a pure tone audiogram (air conduction) and tympanogram were conducted in all subjects. Vestibular function was evaluated by video head impulse test of the horizontal semicircular canal (objectively) and by answering the Dizziness Handicap Inventory (DHI) questionnaire (subjectively). Furthermore, directional hearing was tested calculating the angle detection error as mean square error when noise was presented from −90°, −45°, 0° 45°, and 90° angles. All subjects gave written informed consent to participate in the study, which was approved by the local ethics committee (No. 2016-45) in accordance with the Declaration of Helsinki.

The IBS (Interactive Balance System, neurodata GmbH, Vienna, Austria) consists of four independent force measurement plates for the forefoot and heel (sampling rate: 32 Hz). Based on recordings of vertical pressure variation, general posturographic parameters such as stability or center of gravity changes (product level) can be determined. Furthermore, the force signal over time can be plotted as a frequency spectrogram by fast Fourier transformation. Here, specific frequency bands correspond to the respective postural subsystem (Table 1): F1 represents the visual [26] and nigrostriatal [23] system, F2–4 the peripheral vestibular [21, 24], F5–6 the somatosensory [21], and F7–8 the cerebellar system [23]. This allows for a differentiated analysis of the activity of components that contribute to postural regulation (process level).

**Table 1:** IBS process and product parameter

**Process parameter**	
*Frequency bands*	*Postural subsystem*
F1 (0.01–0.03 Hz) F2–4 (0.0–0.5 Hz) F5–6 (0.5–1.0 Hz) F7–8 (>1.0 Hz)	Visual and nigrostriatal system
	Peripheral-vestibular system
	Somatosensory system
	Cerebellar system
**Product parameter**	
*Parameter of motor output*	*Description*
Stability indicator (ST)	Root mean square of successive differences of pressure signals; describes the postural stability state; the greater the ST, the greater the instability
Weight distribution index (WDI)	Standard deviation of the weight distribution score based on the four plates (ABCD) assuming equal weight distribution on each plate (25% per plate)
Synchronization (Synch)	Six values describing the relationship of vibration patterns between plates calculated as scalar product; 1000, complete coactivity; −1000, complete compensation; 0, no coactivity or compensation
Forefoot–heel ratio (Heel)	Percentage of load distribution forefoot vs. heel with description of heel loading
Side (Left)	Percentage of load distribution left vs. right with description of left side loading

*IBS* Interactive Balance System

When interpreting the findings, it should be taken into consideration that, except for “left” and “heel”, all parameters are dimensionless. The lower the values (except for “synchronization”), the higher the grade of postural regulation. A more detailed description of the IBS can be found in the works of Friedrich et al. [7], Schwesig et al. [25], and Reinhardt et al. [15].

**Fig. 1:**
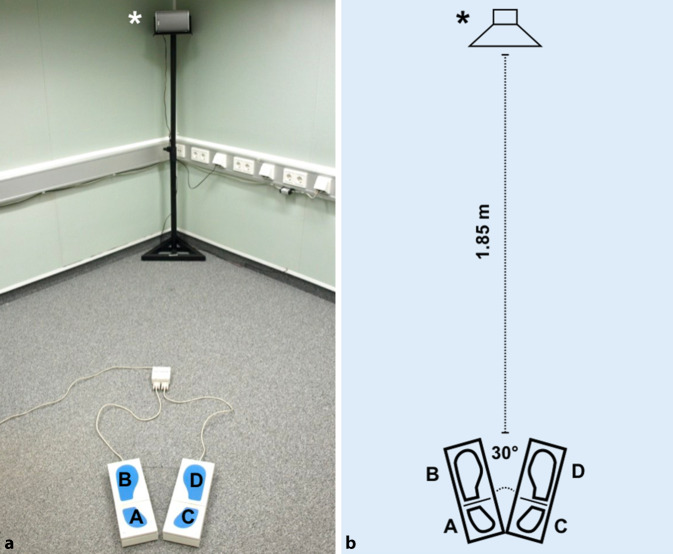
Overview (**a**) and schematic illustration (**b**) of testing setup in a hypoechoic sound-insulated audio booth *Asterisk*: speaker. *A–D:* force-measuring plates of the IBS (Interactive Balance System) measurement system, arranged in an angle of 30° opened toward the front with forefoot (*B*, *D*) and heel area (*A*, *C*)

During testing, the subject stands upright without shoes on two platforms. On each platform a measuring plate for the heel and one for the forefoot is integrated, respectively. The platforms are arranged in an angle of 30° opened anteriorly (Fig. 1). One measurement run consisted of eight testing positions of 32 s, as described in Table 2.

**Table 2:** IBS test positions

Stance position abbreviations	With (+)/without (−) foam pads	Eyes open/closed	Head position
NO	−	Eyes open	Straight
NC	−	Eyes closed	Straight
PO	+	Eyes open	Straight
PC	+	Eyes closed	Straight
HR	−	Eyes closed	Rotated 45° to the right
HL	−	Eyes closed	Rotated 45° to the left
HB	−	Eyes closed	Head up (dorsi-flexed)
HF	−	Eyes closed	Head down (ventro-flexed)

*IBS* Interactive Balance System

All measurements were conducted in a hypoechoic, sound-insulated auditory booth (DIN ISO 825, reverberation time <0.35 s, Industrial Acoustics Company GmbH, Niederkrüchten, Germany). Each test sequence with eight measurements was performed in quiet, with ear plugs (3M, E‑A‑R Classic, noise attenuation [SNR, single number rating] = 28 dB) and with noise (Fastl noise [6], frequency band: 40 Hz bis 20 kHz) presented by a frontal speaker (Canton XL.3, Canton Elektronik GmbH & Co.KG, Weilrod, Germany) at 1.85 m distance, adjusted to the subject’s ear level (Fig. 1). Test conditions alternated in a pseudo-randomized order. With the aim of recording subjective aspects, all subjects had to answer the following two questions at the end of testing:

1. How did, subjectively, the noise influence your sense of balance (answer options: improved, deteriorated, no influence)?
2. Under which condition did you feel you achieved a better testing result (answer options: with noise, without noise, no difference)?

### Statistical analysis

Statistical analysis was performed using SPSS Statistics Version 25.0 for Windows (IBM Co., Armonk, NY, USA). Comparison of means between the two conditions plugged and with noise was conducted by variance analysis (general linear model). The level of significance was set at *p* < 0.0056 (*p* < 0.05/9) after Bonferroni correction or for η_p_^2^ ≥ 0.10 as an indicator of clinical relevance [16]. While the *p* value determines the significance, partial eta squared (η_p_^2^) as an effect size measure allows the evaluation of the clinical relevance.

## Results

### Demographics

A total of 30 healthy subjects were included in the study (mean age: 30.2 ± 11.2 years, range: 19–62 years old; male: *n* = 16, female: *n* = 14; BMI: 22.7 ± 2.88 kg/m^2^). All subjects showed normal hearing based on DIN ISO 7029 (4PTA_0.5–4 kHz_) and normal otoscopy findings. Tympanometry and video head impulse testing results yielded normal values. The mean DHI score was at 0.4 ± 1.1 and directional hearing showed an angle detection error of 0.58°± 2.25.

### Results of IBS measurements

There were no significant (*p* < 0.0056) differences between the two conditions, although clinically relevant differences were found (η_p_^2^ ≥ 0.10): Comparing the foot plate measures between the noise and plugged conditions, we observed in the condition with noise a relevant (η_p_^2^ ≥ 0.10) reduction of postural regulation in the frequency bands F1 (visual and nigrostriatal system, η_p_^2^ = 0.122) and F2–4 (peripheral vestibular system, η_p_^2^ = 0.125), based on the respective mean value of all test positions. On the product level, the parameter WDI (weight distribution index) showed a relevant increase with noise (η_p_^2^ = 0.159). Regarding the postural stability that is represented in the ST parameter, no change was detected between the two auditory conditions (Table 3).

**Table 3:** Interactive Balance System—descriptive comparison of two examinations (mean ± SD, *n* = 30) and analysis of variance for setting *with and without noise *based on the mean values of all positions

Parameter	Descriptive statistics		Variance analysis		Effect size
	With noise (MW ± SD)	Plugged (MW ± SD)	*p*	η_p_^2^	*d*
F1	15.8 ± 5.61	14.5 ± 4.21	0.054	**0.122**	**0.37**
F2–4	8.44 ± 1.99	8.17 ± 1.79	0.051	**0.125**	**0.38**
F5–6	3.64 ± 0.76	3.67 ± 0.80	0.627	0.008	0.09
F7–8	0.66 ± 0.16	0.64 ± 0.15	0.259	0.044	0.22
ST	20.2 ± 4.80	20.2 ± 4.77	0.995	0.000	0
WDI	5.55 ± 1.70	5.12 ± 1.64	0.026	**0.159**	**0.44**
Synch	567 ± 105	566 ± 110	0.932	0.000	0
Heel (%)	46.0 ± 7.79	46.4 ± 7.04	0.627	0.008	0.09
Left (%)	50.5 ± 2.49	50.1 ± 2.20	0.071	**0.108**	**0.35**

Significance was set at *p* < 0.05 or η_p_^2^ ≥ 0.10 and is marked in bold

*F* frequency band, *ST* stability indicator, *WDI* weight distribution index, *Synch* synchronization, *Heel* forefoot–heel ratio, *Left* side loading distribution

### Individual results

Based on the quotient of the ST values between the condition with noise and the plugged condition, an improvement of postural stability in the condition with noise (Q < 0.95) was seen in 30% (9/30), while 40% (12/30) showed no difference (Q: 0.95–1.05) and the other 30% (9/30) displayed deterioration in the condition with noise (Q > 1.05).

### Subjective impression

With regard to the subjective evaluation, in question 1, 40% (12/30) of participants answered that the noise improved their balance, another 40% (12/30) reported no influence, and 20% (6/30) indicated a deterioration. In question 2, 50% (15/30) of participants had the impression they achieved a better testing result with noise, 20% (6/30) felt no difference, and 30% (9/30) reported a better result without noise.

## Discussion

In this study, the influence of hearing on postural regulation and stability was investigated in healthy, normal-hearing subjects using a footplate measurement system. As a result, we observed a shifting of the activity of postural subsystems under auditory input while no difference was seen with regard to the stability indicator (ST).

To date, different approaches have been used to investigate the relationship between hearing and balance including mobile and static measurement methods. The posturographic measurement method used in this study (IBS) is generally comparable to other established footplate measurement systems that have been used to evaluate postural stability, as far as the previously described product level is concerned. Here, the stability indicator (ST) represents postural stability and correlates essentially with parameters of other measurement systems such as sway area, sway distance, or sway intensity described in the literature [7].

Previous trials with methods based on footplate measurement systems mainly report a positive influence of auditory cues on stability:

In the work of Ross et al. [17], a reduced variability of body sway was seen in 19 healthy participants under the presentation of white nose by headphones. Measurements were conducted by means of a center-of-gravity-based footplate measurement system. This effect could also be seen with the same method in an elderly population [18].

Gandemer et al. [8] describe a reduction of body sway on a footplate measurement system under the presentation of rotatory auditory cues compared with a condition with a static sound source or a condition in silence (*n* = 20). In another work, Gandemer et al. [9] investigated in two experiments with 35 healthy participants (1) the influence of different static sound sources in an anechoic and a normal room and (2) with the presentation of multiple three-dimensional sound effects in a hyperechoic environment. In summary, the authors found a reduction of body sway that became more obvious the richer the auditory environment that was presented. This was explained with a model of a spatial auditory map.

In a large study, Vitkovic et al. [33] demonstrated a positive effect of auditory cues, especially rotatory cues on postural stability in normal-hearing volunteers (*n* = 50), in patients with hearing impairment (*n* = 28), and in patients with vestibular dysfunction (*n* = 19). Measurements were made on a Nintendo Wii Balance Board.

Another investigation using a footplate measurement system was conducted by Stevens et al. [31]. There, 18 participants, including six with balance deficits, were tested in different auditory, visual, and proprioceptive conditions. The auditory environment was generated by four speakers positioned in crosswise direction. A significant reduction of body sway with sound exposure was reported, especially in patients with balance disorders.

In a study published several years ago and using a footplate measurement system with the Romberg test, Easton et al. [5] described a reduction of body sway when two laterally positioned sound sources were presented, which was not the case with a single sound source. However, it must be taken into account that lateral sound sources were placed only at a distance of a few centimeters from the pinna. Xu et al. [35] investigated the frequency-specific influence of music on balance in 110 healthy volunteers and reported an improvement of postural control at 100 Hz.

While the aforementioned works generally report a benefit of auditory cues on postural stability, Palm et al. [13] could find no significant advantage of auditory input (music via headphones) in 23 healthy participants compared with visual and proprioceptive situations.

Another study that investigated body sway based on a footplate measurement system in 14 healthy participants with and without ear protection under posterior presentation of white noise also could not demonstrate an influence of hearing on postural stability [12]. Also in the study of Azevedo et al. [2], where the frequency-specific influence of sound on postural control in 20 healthy volunteers was examined, no difference could be seen.

In a study with a focus on the “affective” quality of the presented noise, Chen et al. [4] described increased sway in the anterior–posterior direction during spatial presentation of unpleasant noise, while no difference could be seen in comfortable and neutral sounds. Furthermore, Park et al. [14] reported a deterioration of the center-of-gravity-based sway in higher frequencies, and in the study by Tanaka et al. [32], rotating sound presented through headphones led to increased sway in older people.

Regarding postural stability (ST), no difference was seen between the conditions with noise and plugged in the present work. This is consistent with the results of Azevedo et al. [2], Maheu et al. [12], and Palm et al. [13], who measured no benefit by auditory input. Easton et al. [5] could also show no benefit of the spatial presentation of one frontal sound source compared with stereo sound. This seems to be a possible explanatory approach for the results of this study: Here, noise was presented through a spatial sound source in frontal position, while in most of the studies that described a positive effect, two or more rotating sound sources were used. This is consistent with the findings of Gandemer et al. [9], who conclude that postural stability benefits more from auditory input as richer or more complex auditory environments are used. This is not the case in this study with a single sound source in a hypoechoic environment. It is possible that the benefit of auditory input is clearer in mobile tasks that require a complex interaction of postural subsystems and a continuous scanning of one’s own position in space, as described in a previous study in the Unterberger (Fukuda) stepping test [28].

Furthermore, on the product level, we could see a higher weight distribution index in the condition with noise (WDI: 5.55) than in the plugged condition (WDI: 5.12).

Even if the values remained within the reference range [25], we can see that these differences are indicators of a weight redistribution under presentation of auditory cues.

With regard to the process levels that offer insights into working mechanisms of postural subsystems, we saw in the analyses of the frequency ranges an effect in the frequency bands F1 and F2–4, which indicated a reduction of postural regulation in the visual and nigrostriatal as well as the vestibular subsystems under the presentation of auditory input.

These findings are important in that we could obtain information about reweighting mechanisms of postural subsystems. The visual and the vestibular axis showed reduced activity under auditory input, which indicated a reduced postural regulation capacity in these domains. This seems to follow a compensation mechanism, because in total we could see no influence on the postural stability (ST) at the product level. Somatosensory and cerebellar frequencies were not affected. Maheu et al. [12], too, described a sensory redistribution as an increase of weighting of the visual component in the absence of sound that could not be seen at the somatosensory level. In this way, our results support this study.

Sensory reweighting mechanisms have already been described and investigated elsewhere [1]: Baltes and Baltes [3] explain in the universally applicable model of selective optimization with compensation—which can be applied to the postural system—a compensatory redistribution of resources to achieve an optimized functionality.

Regarding the subjective impression of hearing on balance, interindividual results fluctuate considerably. Even though, depending on the question, 40% of subjects reported an improved sense of balance with noise and 50% felt they had achieved a better testing result with noise, 20% reported a deterioration with noise and 30% reported a better result without noise. This is probably related to the character of the noise. Fastl noise [6] that was used in this study is similar to human language in regard to its spectral distribution and its temporal envelope fluctuations and it has the advantage of not affecting cognition.

However, it seems to have been experienced as unpleasant by some individuals, which can also have a negative effect on postural control. This was demonstrated in the study of Chen et al. [4]: The authors reported an increased sway on a footplate with unpleasant noises compared with neutral or pleasant noises.

## Practical conclusion

- In this study, we showed an activity shift of postural subsystems with auditory input and gained insights into audiovestibular interaction mechanisms.
- How much auditory input influences postural control seems to depend on several factors such as the quality and quantity of the auditory environment and its subjective effect as well as on the complexity and dynamics of the measurement procedure, which should be investigated in further studies.
